# Study protocol of the multi-centre, randomised controlled trial of the Frankfurt Early Intervention Programme A-FFIP versus early intervention as usual for toddlers and preschool children with Autism Spectrum Disorder (A-FFIP study)

**DOI:** 10.1186/s13063-019-3881-7

**Published:** 2020-02-24

**Authors:** Janina Kitzerow, Matthes Hackbusch, Katrin Jensen, Meinhard Kieser, Michele Noterdaeme, Ulrike Fröhlich, Regina Taurines, Julia Geißler, Nicole Wolff, Veit Roessner, Nico Bast, Karoline Teufel, Ziyon Kim, Christine M. Freitag

**Affiliations:** 1 Department of Child and Adolescent Psychiatry, Psychosomatics and Psychotherapy, Autism Therapy and Research Centre of Excellence, University Hospital Frankfurt Goethe University, Deutschordenstr. 50, 60528 Frankfurt am Main, Germany; 2 Institute of Medical Biometry and Informatics (IMBI), University Hospital Heidelberg, Im Neuenheimer Feld 130.3, 69120 Heidelberg, Germany; 3 Department of Child and Adolescent Psychiatry and Psychotherapy, Josefinum Augsburg, Kapellenstrasse 30, 86154 Augsburg, Germany; 4 Center of Mental Health, Department of Child and Adolescent Psychiatry, Psychosomatics and Psychotherapy, University Hospital Würzburg, Margarete-Höppel-Platz 1, 97080 Würzburg, Germany; 5 Department of Child and Adolescent Psychiatry, Medical Faculty Carl Gustav Carus, Technische Universitaet Dresden, Fetscherstr. 74, 01307 Dresden, Germany

**Keywords:** Autism spectrum disorder, NDBI, ASD-specific, Early intervention, A-FFIP, Randomised trial

## Abstract

**Background:**

Naturalistic developmental behavioural interventions (NDBI) have been shown to improve autism-specific symptoms in young children with Autism Spectrum Disorder (ASD). NDBI approaches, such as the ASD-specific Frankfurt Early Intervention Programme for ASD (A-FFIP), are based on ASD-specific developmental and learning aspects. A-FFIP is a low-intensity intervention which can easily be implemented in the local health care/social welfare system. The aim of the present study is to establish 1-year efficacy of the manualised early intervention programme A-FFIP in toddlers and preschool children with ASD. It is hypothesised that A-FFIP will result in improved ASD-specific symptoms compared to early intervention as usual (EIAU). Child- and family-specific secondary outcomes, as well as moderators and mediators of outcome, will be explored.

**Methods/design:**

A prospective, multi-centre, parallel-group, randomised controlled, phase-III trial comparing A-FFIP versus EIAU. A total of 134 children (A-FFIP: 67, EIAU: 67) aged 24–66 months at baseline assessment meeting the criteria for ASD (*DSM-5*) will be included. The primary outcome is the absolute change of the total score of the Brief Observation of Social Communication Change (BOSCC-AT) between baseline (T2) and 1-year follow-up (T6). The treatment effect will be tested, adjusted for relevant covariates applying a mixed model for repeated measures. Secondary outcomes are BOSCC social communication and repetitive-behaviour scores, single ASD symptoms, language, cognition, psychopathology, parental well-being and family quality of life. Predictors, moderators and mediating mechanisms will be explored.

**Discussion:**

If efficacy of the manualised A-FFIP early intervention is established, the current study has the potential to change clinical practice strongly towards the implementation of a low-intensity, evidence-based, natural early intervention in ASD. Early intervention in ASD requires specialist training, which subsequently needs to be developed or included into current training curricula.

**Trial registration:**

German Registry for Clinical Trials (Deutscher Register Klinischer Studien, DRKS); ID: 00016330. Retrospectively registered on 4 January 2019. URL: https://www.drks.de/drks_web/navigate.do?navigationId=trial.HTML&TRIAL_ID=DRKS00016330.

## Background

Autism spectrum disorder (ASD; *Diagnostic and Statistical Manual of Mental Disorders, version 5 (DSM-*5)) is a chronic, pervasive, developmental disorder with a prevalence of ~ 1%, a male: female ratio of ~ 4:1, and a high burden of disease for the affected individuals and their families [1]. Lifetime health economic costs have been estimated for the UK and the US at US$1.4–2.2 million [2]. To date, just 5–10% of adult ASD individuals are living independently in Europe [3]. It is likely, that effective early intervention will decrease long-term costs and improve outcome [4].

Toddlers and preschool-aged children with ASD show severe impairments in many developmental areas, such as visuo-motor abilities, attentional control, joint attention, imitation, social orientation and motivation, social cognition, play, abstract and concept formation, communication and language, emotion regulation and executive function [5–7]. These ultimately result in chronically impaired language, social communication and interaction with peers and adults, which are predictors of adult outcome in ASD [8]. To improve long-term outcomes, thus, developmentally based early intervention needs to be studied for efficacy on the child’s core ASD symptoms as well as on other important developmental areas such as language, cognition and behaviour.

Early intervention in ASD differs with regard to the underlying developmental theory, treatment targets, therapeutic methods, involvement of parents, and intervention intensity. Recent comprehensive systematic reviews [9, 10] agree that the evidence base of any treatment is at maximum moderate, and improvement of core autistic symptoms has rarely been studied. Many of the studied early intervention programmes for toddlers and preschool-aged children are either complex, targeting a broad range of areas with high intensity (> 10, up to 40 h/week work with therapists) [11, 12], or are highly specific with low intensity, targeting core developmental aspects, such as joint attention and symbolic play [13], parent-child interaction [14] or language abilities [15].

High-intensity, therapist-centred, discrete trial trainings (DTT) were studied first [12]. The studies were mostly of low quality, and showed medium to large effects on measured Intelligence Quotient (IQ), but no improvement of core autistic symptoms or behaviour problems. Over time, this approach has been developed further into various new ‘Naturalistic Developmental Behavioural Interventions’, so called NDBIs, in which the behavioural basis has extended by more natural and developmentally relevant components. NDBIs are characterised by naturalistic settings, shared control between child/therapist, natural contingencies, and a variety of behavioural strategies to teach developmentally appropriate, prerequisite skills [16]. In addition, they are including results of recent developmental psychological findings on early development in autism to teach and practise core basic skills, which are impaired in many children with ASD. NDBIs have been shown to increase the child’s motivation to learn, and focus on the integration of knowledge and skills as well as generalisation across different developmental areas [16]. A growing evidence base for NDBIs has been generated, and a recent meta-analysis, including clinical as well as randomised controlled trials (RCTs), reported large effects on improvement of social engagement, medium gains in cognitive development and reduction of core ASD symptoms [17].

One- and 2-year efficacy, especially on improvement of cognitive skills, has been shown by a randomised controlled trial (RCT) on the Early Start Denver Model (ESDM), which is a high-intensity, complex early intervention programme based on a combination of DTT and NDBI methods, the latter focussing on parental synchrony and reciprocity [11]. Again, no improvement of ASD-specific symptoms (Autism Diagnostic Observation Schedule (ADOS) severity score) was observed after 1 and 2 years of intervention compared to treatment as usual with a similar intensity. In a 6-year follow-up study (81% of the original sample), however, long-term improvement of ASD-specific symptoms was reported [18].

Several low-intensity NDBI programmes targeting specific ASD-relevant developmental impairments, such as joint engagement, joint attention, play, imitation, parent-child interaction and language abilities were studied by various RCTs [13–15, 19–22]. All the interventions resulted in clear improvements of the trained abilities, but more distant outcomes often were not assessed. In a secondary analysis, one study on parent-child interaction training showed a medium effect on core ASD symptoms after 13 months, which was maintained at 5-year follow up [23].

No effective, low-intensity, complex NDBI targeting a broad range of developmental areas is available to date. Here, we propose to study a new, complex, manualised, comprehensive and individualised, low-intensity NDBI programme, A-FFIP ([5], more details are provided under the ‘Treatment principles’ section).

A-FFIP usually is provided over a period of 1–3 years before starting school-based education. Pre-post pilot data showed medium effects after 1 year on ASD-specific symptoms [24], language and cognition [25, 26]. Data from a matched, observer-blind case-control study (2 x *N* = 20) confirmed a medium effect size (*ƞ*^2^ = .087; 95% CI .00–.16) of ASD-specific improvement measured by the ADOS severity score compared to early intervention as usual (EIAU) [27].

In addition to establishing efficacy with regard to different outcomes, some early intervention studies on parent trainings explored possible treatment mechanisms (‘why?’). Effectively trained parental behaviour towards the child (parental synchrony [28], parental responsiveness [29], parental mirrored pacing [30]) resulted in increased child’s initiations and joint engagement during parent-child interactive play in three studies [28–30] and ultimately improved ASD-specific symptoms in two studies [28, 29]. Treatment mechanisms of therapist-mediated early intervention have not yet been studied. Possible advantages of therapist- compared to parent-mediated intervention are a faster and more specific gain in pivotal skills by targeted exercises, which, in turn, may influence parental behaviours positively. In addition, mechanisms which go beyond social interaction and social motivation have not been studied, despite the clear notion of additional impairments in ASD going beyond the ‘social-first’ hypothesis in ASD [5, 7].

Given the high variability in outcomes in all early intervention trials, predictors (main effect) and moderators (time × treatment interaction) of treatment outcome (‘for whom’) also need to be studied to be able to individually select specific interventions in the future [31]. A recent review has summarised the following possible predictors and moderators of intervention outcome in ASD: child’s pre-treatment cognitive, adaptive, language, joint-attention and play abilities, and maternal educational level [32]. A longitudinal study on developmental trajectories in preschool-aged children indicated sex and age at diagnosis as additional possible moderators [33].

Recent innovative eye-tracking and body-movement analysis studies aim at objectively measuring sensory perception [34], visual orientating preferences [35, 36] and imitation abilities [37]. Perception, attention, and imitation impairments are likely to underlie core autistic symptoms [7]. In preschool children with ASD, attenuated pupil dilatation to emotion-expressing faces was associated with autistic symptoms [38]. Attenuated visual orientating to social cues (i.e. social attention) is a well-replicated finding in children with ASD [39]. Visual orientating preference for biological motion in preschoolers with ASD predicted symptom reduction after 1 year [36]. In a functional magnetic resonance imaging (fMRI) study with 6-year-old children, pre-treatment levels of neural activity in response to biological versus scrambled motion in four distinct brain regions predicted intervention outcome of a 16-week NDBI (7 h/week) [40]. Eye-tracking and body-movement analysis are feasible and largely tolerated by preschool children with ASD [41]. However, no study applied eye-tracking or objective movement parameters as predictors of outcome in an intervention study.

Limitations of previous studies on complex and targeted NDBI approaches are (1) the lack of a change-sensitive outcome measure capturing core autistic symptoms in a blinded fashion [42], (2) the focus on techniques instead of individualised treatment targets [6, 43] and (3) the lack of complex, low-intensity programmes, which are cost-effective and can be implemented in the community setting [6].

To overcome these limitations, (1) the Brief Observation of Social Communication Change (BOSCC-AT) is used as primary outcome in the current study. This new, ASD-specific, video-based coding system can be obtained by blinded raters and has already shown sensitivity to change in previous studies [24, 44]; (2) A-FFIP is an individualised approach targeting six basic key domains and, additionally, five specific areas of development which are impaired in ASD. Especially training the basic key domains in a developmentally appropriate way is expected to strongly influence child-initiated social learning processes [6]; (3) A-FFIP is a complex, manualised, comprehensive NDBI programme which was conceptualised as a low-intensity approach funded by the German social welfare and health care system within a restricted frame of 2 h/week.

Given the evidence shown above, it is to be expected that A-FFIP, as a complex, low-intensity, manualised, early intervention programme targeting a broad range of ASD-specific, individualised treatment goals and involving parents in the intervention, will result in clinically relevant improvement of ASD-specific behaviour, language and cognition after 1 year.

## Methods/design

### Aims

The primary objective is to establish 1-year efficacy of the A-FFIP on change in ASD-specific symptoms, assessed by a standardised interaction scene of the child with an unknown adult, which is coded in a blinded fashion.

The secondary objectives are (1) To assess A-FFIP effects on child’s cognition, language and behaviour; on parent’s competences, anxiety, depression and stress, and family quality of life; (2) To study child’s and parents’ characteristics as predictors and moderators of outcome; and (3) To explore treatment mechanisms (mediators) related to parents’ and child’s competences and objectively measured behaviour.

### Design

**Fig. 1:**
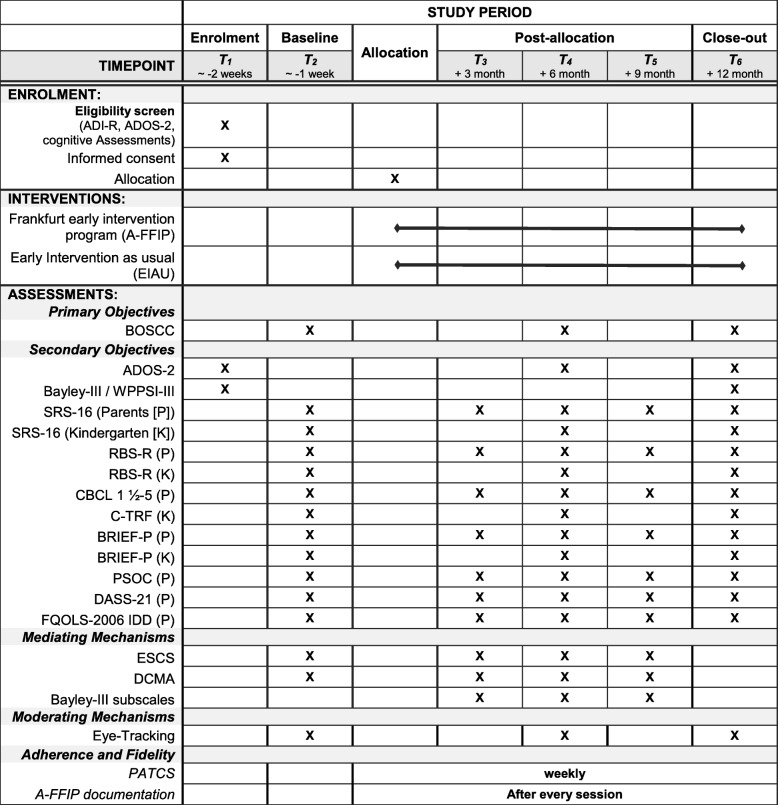
Schedule of assessments. Key: ADI-R Autism Diagnostic Interview - Revised, ADOS-2 Autism Diagnostic Observation Schedule, BOSCC Brief Observation of Social Communication Change, SRS-16 Social Responsiveness Scale – short version, RBS-R Repetitive Behavior Scale – Revised, CBCL 1 ½-5 Child Behavior Checklist 1 ½-5, C-TRF 1 ½-5, BRIEF-P Behavior Rating Inventory of Executive Function-Preschool version, PSOC Parent sense of competence scale, DASS-21 Depression Anxiety and Stress Scale – short form, FQOLS Family quality of Life Survey, ESCS Early Social Communication Scale, DCMA Dyadic Communication Measure for Autism, Bayley-III Bayley Scales of Infant and Toddler Development 3rd Edition, PATCS Parent Adherence to treatment and Competence Scale

The trial is designed as a confirmatory, phase-III, prospective, randomised, multi-centre, controlled, parallel-group study with two treatment arms and six measurement time points. The trial time flow is shown in Fig. 1.

### Setting

The study will take place in a clinical setting at four German study centres which provide a special outpatient service for individuals with ASD (University-based Departments of Child and Adolescent Psychiatry, Psychosomatics, and Psychotherapy at Augsburg, Dresden, Frankfurt and Würzburg). The sponsor is the Goethe University Frankfurt (GU), and the principal investigator is Prof. Dr. CM Freitag, University Hospital Frankfurt at GU. The study-related diagnostic tests for all children (A-FFIP and EIAU) are performed at the four study centres, and are coded in a blinded fashion by independent individuals who are not involved in any intervention. A-FFIP is delivered by two therapists/child at the four study centres, who are not involved in any study-related diagnostic procedures. EIAU is delivered in any local setting outside the four study sites.

### Participants

#### Inclusion criteria

Only children meeting the following criteria are included:

- All subjects must meet *DSM-5* criteria for Autism Spectrum Disorder. Diagnostic assessment is standardised according to *DSM-5* by performing the semi-structured Autism Diagnostic Interview (ADI-R), toddler algorithm with the parents, and the standardised Autism Diagnostic Observation Schedule-2 (ADOS-2) with the child
- Age range: 24–66 months old at T2
- Written informed consent of the legal caretakers of the patient
- Ability to regularly and reliably attend appointments
- Parents ability and willingness to attend at least every fifth therapy session with the child

#### Exclusion criteria

Subjects presenting with any of the following criteria will not be included in the trial:

- Non-verbal Developmental Quotient (DQ)/Intelligence Quotient (IQ) ≤ 30
- Non-verbal mental age ≤ 12 months
- Diagnosed vision or hearing impairments interfering with therapy
- Cerebral palsy
- Chronic neurological disorder
- Unstable epilepsy
- Neurodegenerative disorder
- Rett/Angelman syndrome
- (History of) severe psychosocial deprivation
- Insufficient care by parents
- Attachment disorder
- Institutional upbringing
- Parents not verbally fluent in German and/or unable to read German

#### Recruitment

The majority of the local ASD patients are diagnosed at the four participating study centres. Every ASD patient meeting the inclusion criteria will be invited to participate in the study. Additionally, flyers with information about the study will be provided at other medical and public facilities to reach more interested parents for inclusion. To promote participant retention and complete follow-up in all participants, the individual baseline and endpoint results of the ADOS-2 and the results of the developmental and cognitive tests will be provided and explained to the parents after the completion of the study. The families in the control group are offered participation in the A-FFIP intervention after completion of the study-related assessments after approximately 1 year. In case of any unexpected and unanticipated adverse events (AEs) of the intervention during the study (the assessment of serious adverse events (SAEs) is described under ‘Harms’), parents will be informed about these events prior to participation or during the study. Published research data will be shared with interested families by mail.

#### Withdrawal

Subjects may either withdraw from the intervention, but will stay in the study, or the subjects may totally withdraw from the study. A third option is that due to SAEs or other events, the principal investigator decides that the subject has to withdraw from the intervention. In all cases, the reason for withdrawal must be recorded in the patient’s Case Report Form (CRF) and in the subject’s medical records. In case of (full) withdrawal of a subject at the request of their legal representative, the reasons will be explored as extensively as possible. The subject will be followed up and – if possible – all examinations scheduled for the final trial day will be performed on all subjects and documented.

### Intervention to be investigated

The A-FFIP intervention manual [5] provides the clinical therapist (behaviourally trained psychiatrist/psychologist/social worker) with information on the theoretical background, description of the diagnostic assessments necessary before start of the intervention (including parent interviews), choice of individualised treatment targets, and a detailed description of the target-related specific exercises, including the toy-based training material (for more detailed information in English see attachment 1 of Kitzerow et al. 2019 [27]: https://econtent.hogrefe.com/doi/suppl/10.1024/1422-4917/a000661/suppl_file/1422-4917_a000661_esm1.pdf).

Intensive A-FIPP therapy training for all involved therapists will be done before treating children within the study. The therapy training comprises:

- Individual study of the A-FFIP manual
- Two participations in a 3-day workshop led by the first author of the A-FFIP manual and head of the Autism Intervention Centre at Frankfurt (K Teufel) *or* shadowing a qualified A-FFIP therapist of the Frankfurt group for 1 week
- Practising the implementation of A-FFIP (planning of treatment targets, treatment principles, etc.) with a preschool-aged child with ASD not included in the study; video-documentation of intervention sessions
- Targeted supervision based on these videos, which are rated prior to supervision by an experienced therapist based on the A-FFIP therapy fidelity checklist
- Based on the results of the therapy fidelity achieved by the therapist, the practising and supervision are repeated until a satisfying A-FFIP fidelity score is achieved:
  - *Total score* (range 0–56): 50 points
    *and*
  - *Item scores* (range 0–2): at least 1 point in each item

Continuous training and video-based telephone supervision will be provided by the Frankfurt group to ensure high treatment fidelity of therapists. The on-going supervision per centre comprises:

- Every second session of the first five patients per centre and every eighth session of subsequent patients will be video recorded, sent to the Frankfurt supervision team and rated with the A-FFIP fidelity checklist
- Detailed feedback and supervision is provided by telephone once a month or in case of particular serious manual violations

An additional 2-day workshop once a year led by the first author of the A-FFIP manual with current questions by the therapists, examples from the supervisors and presentation of the current fidelity scores.

#### Setting

Sessions are provided in an outpatient setting at the respective study centre. All treatment rooms are furnished in a standardised way, with standardised and well-arranged play material, ensuring a structured environment providing orientation and a minimum of distraction.

The intervention comprises 2 h of intervention/week with two therapists working with the child. The two-therapist concept is an essential A-FFIP component. While the main therapist interacts with the child, the co-therapist acts as a shadow and prompts the child individually and in a situation-specific way, during the learning of new abilities, and only if necessary. This concept is believed to support the natural learning of the child, because the child will learn the specific skill or task faster, and the interaction with the main interaction partner (therapist/parent) is independent from any prompting efforts. If the child is very advanced and shows interest in social interaction with peers, two therapists will work with two equally advanced children. At least one parent is participating in a minimum of every fifth therapy session for psychoeducation and exercises with their individual child, with the aim of learning to use effective strategies for practising at home. Parents are asked to practise established skills at home in many naturally occurring situations; these tasks are determined by the therapist and the implementation is discussed regularly with the parents. To ensure compliance by supporting parents without increasing pressure, no specific amount of practising hours or situations is expected. Parental adherence and competence is assessed by a weekly questionnaire (PATCS, see section ‘Mediating mechanisms’).

If the child already attends kindergarten, one therapist visits the child’s kindergarten teachers, and (if existing) the personal assistant three times a year for individualised psychoeducation with the aim to support generalisation of the child’s acquired skills at kindergarten. The first appointment is organised directly at the beginning of the intervention.

#### Individualised treatment targets

The concept of A-FFIP is based on the individualised practising of developmentally based and highly specific treatment targets, taking latest research findings on ASD-specific development into account. This has been achieved by developing specific exercises based on developmental psychological science findings. Especially this third aspect is taking on, but also going beyond, current low-intensity NDBIs such as JASPER [13], PACT [14], pivotal response treatment [15] or imitation training [19], which focus on one or two core developmental aspects.

A-FFIP targets six core basic abilities (attentional control, joint attention, imitation, representation, planning, self/other distinction) and five developmental domains (language and communication, interaction and play, emotion regulation, cognition and adaptive behaviour).

For each of the core basic abilities and developmental domains, exercises for beginners, intermediate and advanced learners are provided in the A-FFIP manual. In addition, for each exercise, specific advice for therapists is given on how to correctly execute the exercises and how to teach parents the correct execution. The play material used is highly flexible according to the preferences of the child, increasing the child’s motivation to learn and to interact with the therapist. A-FFIP is an individualised intervention programme that takes the child’s current ability level into account [43, 45]. This rationale is essential due to the heterogeneous strengths and weaknesses of children with ASD in different developmental domains. A-FFIP expects the therapists to choose exercises that are slightly more advanced than the child’s current abilities promoting successful learning. Methods and exercises are highly standardised.

At the start of the A-FFIP intervention, a thorough assessment, based on ADOS-2 and Bayley Scales of Infant and Toddler Development – Third Edition (Bayley-III) results, play sessions with the child and parental report on the child’s behaviour at home, is done to elicit the abilities of the child in each of the basic and specific developmental domains. The collected information from the standardised instruments and from the direct observation is transferred to the detailed A-FFIP intervention targets checklist (for further information see attachment 1 of Kitzerow et al. 2019 [27]), which is the basis for planning the individual relevant interventions goals. Additionally, the parents are asked to complete a list of rewarding activities, and a broad range of positive rewards are tested at the beginning of therapy. Based on the results of this first assessment and parental priority, specific A-FFIP treatment targets are chosen together with the parents (maximum eight targets).

Progress in each area is monitored by the therapist filling in a specific documentation form adjusted to the A-FFIP intervention targets checklist after each session. Once the child has achieved a treatment target, a new target is focussed, which builds on the previous targets. Established skills will be trained in an ongoing fashion in different situations (e.g. at home with parents/siblings, at kindergarten) to assure generalisation.

#### Implementation of specific methods

A-FFIP has been developed on a strict empirical basis; therefore, effective, behaviourally based learning techniques are implemented, especially techniques supporting associative, operant, imitative and social learning (such as prompting, naturally based positive reinforcement, modelling, generalisation), and techniques reducing interfering, aggressive and stereotyped behaviour, such as antecedent- and consequence-based interventions [46] focussing on enhancing the child’s coping skills (e.g. by providing structure and routine). The child’s motivation to learn, play and socially interact is increased by natural reinforcement intrinsic to the child’s interests and activities, by therapist (and parental) synchrony, and by consistent positive reinforcement of self-initiated learning.

### Control intervention: early intervention as usual (EIAU)

For EIAU, individual or group therapy intensity of 1–10 h/week as well as waiting time prior to any intervention onset is allowed. This is representative for the German ASD preschool population, and comparable average intervention intensity per week is expected for both groups with higher variability for EIAU than A-FFIP [47].

### Additional treatments and medication use

In the A-FFIP as well as the EIAU groups, the following additional treatments are allowed: stable psychopharmacotherapy, stable medication for chronic medical conditions not interfering with the therapy, individual speech and language, occupational or physiotherapy, personal support at kindergarten/preschool or family support. Any additional treatment will be documented exactly (kind of intervention, dose, frequency, etc.) and will be compared for non-random distribution between groups in the statistical analysis.

Psychotropic medication will be started or changed at least 4 weeks before randomisation and will remain stable (mg/kg body weight) throughout the intervention (with the exception of dose adjustment to body-weight changes). The following psychotropic medication will be allowed as single or combined treatment: SSRI, other antidepressants, antipsychotic medication, atomoxetine, mood stabilisers. In addition, stable medication for the treatment of chronic conditions, such as allergies, asthma, epilepsy, enuresis, sleeping problems and intermitting medication for acute upper respiratory infections and diarrhoea, will be allowed. Pharmacological treatment will be documented at each time of assessment (T1-T6) and psychotropic medication effects on treatment outcome will be explored in analysing study outcomes.

Children in the Intervention group are permitted to receive any other intervention during the waiting time before the start of A-FFIP.

The following concomitant treatments are not permitted during the trial:

- Additional general or ASD-specific early intervention (in the A-FFIP group)
- Additional parent training (in the A-FFIP group)
- Treatment in a day-care facility or ward of a child psychiatric department (A-FFIP and EIAU group)
- Elimination diets or therapy (A-FFIP and EIAU group)

Parents will be informed about these study requirements prior to inclusion and randomisation. If the clinical necessity arises to start any elimination diet or pharmacotherapy, this will be documented, and the child will be allowed to further participate in the study. If a child needs to be admitted for day-care or inpatient child psychiatric treatment, the child will have to stop participation in the study.

### Outcomes

#### Primary outcome

Change of ASD-specific symptoms is of strong clinical relevance due to long-term effects on adult outcome of the disorder [8, 23]. Therefore, the absolute change of ASD-specific symptoms, measured by the average total score of the Brief Observation of Social Communication Change (BOSCC-AT; latest version from 11 December 2017) between baseline (T2) and 1-year follow-up (T6) is the primary outcome measure. The BOSCC [24, 44, 48] is a new, reliable (inter-rater intraclass correlation coefficient (ICC) = .98), change-sensitive and valid observational measure to study change in social communication and interaction as well as repetitive and stereotyped behaviours in ASD which has been recommended in a recent Health Technology Assessment [42].

#### Other measures - diagnostic assessments

The *ADI-R* is a semi-structured diagnostic interview with the parents, assessing the core autism symptoms in the areas of social interaction, communication and stereotyped behaviour [49, 50].

The *ADOS-2* is an observational; semi-structured and standardised assessment of communication, social interaction, play and restricted and repetitive behaviours. It is the gold standard to use for ASD diagnosis [51–53]. It comprises different modules dependent on age and verbal level.

The *Bayley Scales of Infant and Toddler Development – Third Edition (Bayley-III)* [54] is an international developmental test und will be used for children with a developmental age below or equal to 42 months at the respective time point. The reliability for the German version lies between *r* = .77 and *r* = .89, and construct validity is comparable to the original version. In this study, the cognitive, language and fine-motor subscales will be used.

The *Wechsler Preschool and Primary Scale of Intelligence (WPPSI-III)* [55] is an international multidimensional measure of intelligence for preschool-aged children (3;0–7;2 years), and will be used in children with a developmental age > 42 months. Reliability is high with *r* = .95 for total IQ.

No valid, uniform IQ test covering the entire developmental age of the sample is available in Germany, thus the two different IQ measures with current norms had to be chosen.

#### Secondary outcomes

##### Child - ASD-specific symptoms

The *BOSCC subscales* (social communication BOSCC-SC, restrictive and repetitive behaviour BOSCC-RRB) and single items are examined for a differentiated picture of change in ASD-specific symptoms [24, 44]. Mean single-item scores have shown satisfying inter-rater reliability (ICC = .54–.97) and internal consistency (social communication subscale BOSCC-SC *α* = .83, repetitive behaviour subscale BOSCC-RRB *α* = .41) in a recent version. A revised and updated version of the BOSCC will be used for this study, which is expected to show even higher reliability.

The *ADOS-2 comparison and domain scores* will additionally be used. The comparison score allows the comparison between different time points, independent of the respective module. The ADOS-2 comparison score is based on the ADOS severity score. To compare the subscales Social Affect (SA) and Restricted and Repetitive Behaviours (RRB) calibrated domain scores will be used [56]. The ADOS-2 shows high inter-rater reliability for modules 1–3, with an ICC of .96 for the overall total. Internal consistence varies from *α* = 0.87 to *α* = 0.92 in the SA domain and from *α* = 0.51 to *α* = 0.66 in the RRB domain [51].

The parent- and kindergarten-teacher-rated *Social Responsiveness Scale (SRS)* [57] measures social responsiveness. The internal consistence is high (*α* = .93–.97), and the SRS has shown moderate validity compared to other ASD-specific measures. It has been recommended for use in ASD early intervention trials [42]. We will use the Social Responsiveness Scale – short version (SRS-16) 16-item short version which has been shown to have a high internal consistency and reliably measures change of ASD symptoms in one social-communication domain [58].

The parent- and kindergarten-teacher-rated *Repetitive Behaviour Scale-Revised (RBS-R)* [59] measures ASD-specific repetitive behaviours. These behaviours can substantially interfere with family activities and learning processes. Internal consistency for the subscales is satisfying ranging from *α* = .78 to *α* = .91 [59]. High correlations with the Child Behaviour Checklist (CBCL) and the ADI-R were found, and factor structure and reliability have been replicated for young children with ASD [60, 61]. The German translation and analysis of German norming data of the RSB-R has replicated the factor structure of the original publication (unpublished data, Frankfurt).

##### Child - Cognition and language

Depending on the child’s developmental level a standardised test with current German norms is used to assess cognition and language. Different forms of early intervention in ASD have been shown to improve cognitive and language development [11, 12, 27], which is of long-term relevance, because higher IQ and language level in childhood predicted adult outcomes [8]. The *Bayley-III* [54] or the *WPPSI-III* [55] will be used in relation to the child’s developmental stage (see above, ‘Diagnostic process’).

##### Child - Additional measures

The parent rating form *Child Behaviour Checklist 1½-5 (CBCL1½-5)* [62] and the *Teacher Report Form 1½-5 (C-TRF)* [63] are two of the most widely used valid and reliable measures in clinical research, dimensionally measuring social-emotional or behavioural problems [64]. The CBCL has been recommended as one of 12 most valid instruments assessing outcome in ASD early intervention trials [42].

The *Behaviour Rating Inventory of Executive Function-Preschool Version (BRIEF-P)* [65] is a rating scale assessing executive function (EF) in preschool children (2;0 to 6;11 years). Internal consistency varies from between *α* = .75 and *α* = .96 depending on rater/subscale. Inter-rater reliability for the total score is *r* = .56. Concurrent validity was shown for CBCL subscales. In preschool children with ASD, real-world EF impairments were observed, which were not related to ASD symptoms [66]. Given the relevance of EF problems for adult outcome, change in EF by early intervention is an important outcome [67].

##### Parents and family

The German adaptation of the *Parenting Sense of Competence Scale (PSOC/FSW)* [68] is chosen to measure parental self-efficacy. It has been developed and used in previous DFG projects (Zukunft Familie I, Ha1400/14–1-4). The revised questionnaire has been validated with young children (2.5 to 6.5 years) and shown satisfying internal consistency for mother and father assessments (*α* = .78/.79). Convergent validity with other measures of parental stress and competences has also been confirmed in the validation study [68]. The PSOC has been used in several ASD studies [42].

The *Depression Anxiety and Stress Scales – short form (DASS-21)* [69] is chosen to measure parental mental distress. Internal consistency is high, varying from *α* = .76 to *α* = .91. The validation study has shown good convergent validity to other measures of depression and anxiety (*r* = .59 to *r* = .86). Parental mental distress likely influences parent-child interaction, and may be changed by early intervention [70].

The *Family Quality of Life Survey-2006 ID/DD version (FQOLS-2006-ID/DD)* was developed for the main caregivers of persons with intellectual and developmental disabilities and has been translated into 20 languages, including German [71]. It covers nine domains, of which four are assessed here. A validation study reported high internal consistency (*α* = .85) and concurrent validity (*r* = .63) with another quality of life measure [72]. Improving family quality of life for families with an ASD-affected member is an important goal in autism intervention [73].

### Predictors and moderators

IQ/DQ, BOSCC-AT, gender, ADI-R and both parent‘s educational status will be investigated. The educational status will be classified according to the *International Standard Classification of Education (ISCED)* 2011.

An eye-tracking battery will be applied at T2, T4 and T6. This *eye-tracking battery* includes measures of sensory perception and visual orientating preferences. These tasks include: pupil adaption fixation stability (PAFS), smooth pursuit (SMT), visual oddball (VOT), visual search (VST), non-social versus social reward (NSRT), dynamic emotion expressing faces (DEEF), natural scenes (NST), biological motion visual preference (BMVP) and joint attention task (JAT).

*Spontaneous imitation* is assessed by automated-movement analysis via depth sensor cameras during video-based assessments (ESCS, BOSCC, DCMA, ADOS-2).

*Parental treatment attendance* will be documented weekly by therapists [74].

### Mediating mechanisms

The *Early Social Communication Scales (ESCS)* [75] is a standardised behavioural observation measure of non-verbal communication and social interaction skills that typically emerge in children with a developmental age of 8 to 30 months. For joint attention and social interaction, inter-rater reliability is > .8. The ESCS have been used as a change-sensitive outcome measure of early intervention trials [42, 76].

Bayley-III scales: see above. The cognitive and fine-motor scales will be studied as mediators.

The *Dyadic Communication Measure for Autism (DCMA)* [77] systematically rates quality of dyadic parent-child communication. Inter-rater reliability was ICC = .8 for parent synchrony and ICC = .59 for child initiations. The DCMA has shown good sensitivity to change, and parent synchrony mediated the child’s ASD-specific outcome in a large RCT on parent-mediated early intervention [14, 23, 28].

*Parental treatment adherence and competence* in employing techniques at home will be studied by the A-FFIP-adjusted *Parent Adherence to Treatment and Competence Scale (PATCS)*, which has been used in previous early intervention trials [78]. It is a self-report with four items related to adherence and two items to competence; all rated by a 5-point scale (low to high); Cronbach’s α = .82.

### Procedures

#### Blinding

As the RCT is a behavioural therapy intervention study, blinding of participants, therapists, parents and kindergarten teachers is not possible. To minimise observation and detection bias, blinded trained observers will collect data for all direct observation measures (BOSCC, play session, ESCS, cognition, language) especially for the primary endpoint (BOSCC-AT). The rating will be done by independent coders blind to treatment and randomisation status.

#### Randomisation

After written informed consent and baseline testing for eligibility, patients will be allocated by randomisation in a 1:1 manner to the interventions groups using a centralised, web-based tool (randomizer.at). Block randomisation will be performed for each centre and gender to achieve equal group sizes within these strata.

#### Data collection

All findings including clinical data will be documented anonymous in the subject’s medical record and in the CRF.

The BOSCC will be done by an independent local tester who plays with the child. The BOSCC scores will be rated from video by independent raters located in Frankfurt. BOSCC video data will be stored until about year-2 of data collection. Regular coding will then be done by three to four raters, trained to high inter-rater reliability. Every fifth video will be coded by all coders for measuring ongoing inter-rater reliability. The BOSCC is coded based on a 12-min, semi-standardised videotaped play situation. Two 6-min segments are watched twice while taking notes, and are coded immediately by a standardised scheme. Fifteen items are rated from 0 to 5 according to item-specific decision trees. Mean values from both segments are calculated for each item, and the summary scores are derived: BOSCC-AT (total score, items 1–13; (without item 9)), BOSCC-SC (items 1–8), BOSCC-RRB (items 10–13) and BOSCC single-item scores.

In each centre, every second therapy session of the first five patients and every fifth session of the following patients are videotaped for quality assurance.

#### Data management

The Institute of Medical Biometry and Informatics (IMBI) is responsible for the data management using a validated system. In order to ensure that the database reproduces the CRFs correctly, a double entry of data is performed by two different persons. A query process based on a beforehand-specified data-validation plan is established. Any entry and correction in the study database will be reported automatically in an audit file. All data management activities will be done according to the current Standard Operating Procedures (SOPs) of the IMBI.

### Statistical analysis

#### Sample size calculation

Sample size calculation refers to the primary outcome measure absolute change of BOSCC-AT between baseline (T2) and 1-year follow-up (T6). An age- and DQ-matched, but not randomised, observer-blind, case-control study of 2 x *N* = 20 children with ASD aged 3.2–7.9 years and an IQ/DQ of 37–134 at the start of intervention resulted in an A-FFIP 1-year effect size of 0.61 for the ADOS-severity score [27]. In a pre-post sample (*N* = 21) aged 3.8–5.8 years with a lower IQ/DQ 37–108, the 1-year pre-post effect size of the BOSCC-AT was 0.63 [24]. ADOS items are the basis for the ADOS-severity score and the BOSCC [48]. The BOSCC shows a higher number of items coded on a 6-point scale compared to the 2-point scale of the ADOS, resulting in higher variability capturing more subtle changes (range BOSCC-AT 0–60 versus range ADOS 1–10). For the BOSCC-AT, a more moderate, clinically meaningful, effect size of 0.55 is expected. With a significant level of *α* = 5% (two-sided) and a power of 1 − *β* = 80%, a sample size of 106 (2 × 53) is required to detect an effect size of 0.55 with the two-sample *t* test (ADDPLAN, version 6.1.1). Considering a drop-out rate of 20%, 134 (2 × 67) patients will be randomised to the treatment groups. It can be expected that including covariates in the confirmatory analysis will increase power compared to the *t* test.

#### Definition of analysis sets

Patients will be allocated to the different population sets (per-protocol set, full analysis set, according to the intention-to-treat (ITT) principle and safety set). A final definition of the analysis sets is in given the statistical analysis, which is finalised prior to the analysis.

#### Statistical methods

The primary analysis will be conducted based on the ITT population. Let *μ* denote the unknown true mean change of the BOSCC-AT between T2 and T6. The null hypotheses:

H_0_: *μ*_A-FFIP_ = *μ*_EIAU_ is tested against the alternative: H_1_: *μ*_A-FFIP_ ≠ *μ*_EIAU_.

The confirmatory test for treatment-group difference with respect to the primary efficacy endpoint will be done applying a mixed model for repeated measures (MMRM) approach [79] modelling the difference to baseline including the fixed-effects baseline BOSCC-AT, chronological age, treatment group, time, and treatment-group-by-time interaction; centre will be included as random effect. The time points of measurement are T2 (baseline), T4 (weeks 26–27) and T6 (weeks 52–54). The significance test for treatment-group difference will be based on least-squares means using a significant level of *α* = 5% (two-sided).

No missing data are expected for the covariates. Missing values for the primary outcome measure are dealt with as follows in the MMRM approach: The likelihood-based approach jointly models all actual observations without imputing missing data but using the within-patient correlation structure to provide information about the unobserved post-baseline primary outcomes and gives reliable results under the missing data at random (MAR) assumption. The MMRM approach shows favourable characteristics in terms of type-I error rate, power and bias of estimates as compared to alternative methods dealing with missing values, such as last-observation-carried-forward (LOCF) [79–81], even in the presence of a drop-out rate of 20% [82].

Data quality and homogeneity of intervention groups at baseline will be evaluated. All secondary endpoints will be analysed descriptively, using appropriate statistical methods.

#### Additional analyses

As a sensitivity analysis the primary endpoint will be evaluated based on the per-protocol set of patients without major protocol violations. Additional sensitivity analyses will be done using LOCF and complete case analysis via analysis of covariance (ANCOVA) for repeated measurements. Safety analysis includes calculation and comparison of frequencies and rates of (S)AEs.

*Analysis of predictors and moderators*: in an exploratory fashion, implementing the above–mentioned MMRM approach, including the pre-specified variables as main or interaction effects in the respective models. For each pre-specified variable an own model will be calculated.

*Treatment mechanism* on the primary efficacy endpoint will be investigated following the mediation analysis approach described in Pickles et al. 2015 [28].

All analyses will be done using SAS® version 9.4, except for utilising Mplus software for the investigation of treatment mechanism.

## Discussion

The present randomised controlled, multi-centre, parallel-group trial including an active control group aims at establishing efficacy of the manualised A-FFIP early intervention programme for toddlers and preschool-aged children with ASD on ASD-specific symptom change after 1 year. As stated in the introduction, A-FFIP has been developed on a strict empirical basis as a low-intensity, comprehensive, individualised early intervention, which is unique in the field of early intervention in ASD.

The design of the study addresses a number of relevant issues in ASD-specific early intervention research and also aims to overcome limitations of previous studies, i.e. study quality [83], questionable use of primary outcome measures [42] and missing moderator analyses [32].

Study quality is ensured by a number of specific procedures. First, intensive training in the relevant diagnostic instruments, such as ADOS-2, BOSCC, ESCS and DCMA, will be provided by the Frankfurt study group. Video-based ratings prior to study start will be done to ensure full objectivity of the testing procedure. Similarly, an intensive training in the manualised A-FFIP intervention is provided by the first author of A-FFIP and long-standing A-FFIP experts in the Frankfurt group. The training is accompanied by ongoing video-based feedback prior to, and during, intervention. Therapists need to meet pre-specified criteria regarding their therapy-related competencies before being allowed to treat the children within the study and will be supervised regularly during the study period. Most high-quality studies in early intervention, such as PACT [14, 84] and ESDM [11], reported on their training procedures, which are, overall, comparable with the one implemented in the present study.

Second, during the study, the primary and secondary video-based outcome measures (BOSCC, DCMA, ESCS, ADOS-2) are rated by a small Frankfurt-based team, with established high inter-rater reliability and ongoing consensus ratings, to reduce the risk of measurement errors. The diagnostic team leader has received training on the BOSCC and the DCMA by the developer of the regarding instruments, before. This procedure has also been reported in similar intervention studies [44, 84, 85].

Third, as recommended by the International Council for Harmonisation of Technical Requirements for Pharmaceuticals for Human Use – Good Clinical Practice (ICH-GCP) [86] data management, study monitoring and statistical analyses are done by independent institutions, specialised in their respective field. The ICH-GCP standard has rarely been implemented in psychotherapy trials, despite its major role in European pharmaceutical trials.

By choosing the BOSCC as the primary outcome measure, changes in ASD-specific behaviour are investigated by a change-sensitive instrument. The BOSCC will be done in a standardised and blinded fashion [24, 44]. The chosen BOSCC testing situation with a foreign person will also add information about generalisation of acquired competencies. Previous studies have chosen different primary outcome measures. In most studies, in which distant outcome measures were used, especially cognitive (i.e. IQ measures), language or non-blinded measures of adaptive abilities (i.e. Vineland Adaptive Behaviour Scales, VABS) were chosen as primary outcome [12, 42, 87].

Thus, many early intervention studies do not give any information on the central question of intervention in ASD, namely the improvement of core ASD symptoms. In addition, most studies did not use blinded outcome measures, especially such studies, which have implemented parent-based questionnaires as primary outcome measure, such as the VABS.

Only one multi-centre RCT has used the blinded ADOS as primary outcome to describe change in core ASD symptoms [14, 84]. In the primary analysis, no change of core ASD symptoms was observed. This likely is due to the character of the ADOS, which was not designed to measure behaviour change over time, but as a diagnostic instrument [42]. The BOSCC has been developed based on the ADOS, but with a revised scoring system and additional items, thus showing higher measurement variability and sensitivity to change [48]. It is currently also implemented in several studies internationally [84, 85].

Additional, the secondary outcome measures of the present study will allow the detection of change in the child’s cognitive and, language development, additional internalising and externalising psychopathological symptoms, and A-FIPPs’ effects on parent’s behaviours, family well-being and quality of life.

Using these various secondary outcomes allows a direct comparison with other studies, such as cognitive and language development within the ESDM study [11], or gain in parental self-efficacy (questionnaire) and parental synchrony (DCMA) within the PACT study [84]. In addition, several family related measures have been added which have rarely been reported in previous studies, meeting the expectation of the parents on relevant outcome measures [42].

The unique, low-intensity, but complex and comprehensive NDBI concept of A-FFIP also adds valuable information to the discussion about needed treatment intensity for children with ASD [16, 88]. Low-intensity approaches can be delivered to more families compared to high-intensity approaches, and might be more cost-effective than high-intensity approaches. Additionally, recent studies have shown that some comprehensive, high-intensity approaches did not lead to change in core ASD symptoms [12], but focussed, low-intensity approaches (as in the PACT study [14, 23]) achieved long-time changes in ASD-specific behaviour. Future studies may focus on the clinical relevance of the respective changes, and efficacy and cost-effectiveness studies should directly compare low- and high-intensity approaches.

Regarding predictors and moderators of intervention outcome, most frequently age, IQ, or ASD-specific symptoms prior to the start of the intervention, have been studied as possible predictors or moderators of treatment outcome [32]. This also will be done in the present study. In addition, the objective measures implemented, such as eye-tracking and objective movement parameters are unique, and will provide important additional information on ‘what works for whom’ [36]. This may also allow us to partly explain the variability in outcomes of early intervention trials.

The analysis of mediators will allow an exploratory therapy process analysis. Child-specific as well as parent- and therapist-specific characteristics will be explored as mediators, such as the child’s learning curve on essential abilities as joint attention. Therapist fidelity is regularly assessed which allows us to specifically elicit the role of the therapist-related competencies on intervention outcome. Parental treatment adherence and competence are assessed by a weekly questionnaire (PATCS) filled in by the parents, on their self-rated ability to implement the learned strategies in their daily routine. A-FFIP is conceptualised as a low-intensity, therapist-delivered programme, in which parents are regularly supported to encourage the generalisation of new, individually meaningful and developmentally appropriate skills in daily, natural routines. The extent of parental involvement is adjusted to the individual’s familiar needs and resources. In addition, child-specific psychoeducation is provided for kindergarten teachers. Parents and kindergarten teachers are not expected to do particular exercises with the child. Future studies might focus on more objective fidelity measures of the parental implementation of intervention methods and targets at home. In the current study, the parent rating questionnaire will provide data on the parent’s sense of satisfaction, competence and intensity using A-FFIP strategies at home. This information will again be explored as a possible mediator of therapy outcome.

If efficacy is shown, additional expected positive effects by the trial will be: (1) Improvement of care in underserved areas in Germany (such as Bavaria, Saxony); (2) Alerting many different professionals working in early intervention in ASD to effective methods and (3) Establishing an effective training and supervision method provided to professionals to achieve high treatment fidelity of evidence-based early intervention. Thus, the current study may change clinical practice strongly towards the implementation of evidence-based early intervention methods in ASD in Germany. Because A-FFIP is delivered through two therapists for a 2-h/week intervention it may also be applicable in other areas with comparable public health systems. Therefore, the study results should be of great impact for many ASD underserved regions.

### Trial status

Protocol version V2 from 4 October 2018. Recruitment started on 18 April 2018. Recruitment is planned to be completed in 2019.

### Monitoring

All monitoring institutions and members are independent of the sponsor and competing interests.

### Trial monitoring and auditing

Clinical monitoring will be performed by the coordination centre for clinical trials (KKS) Heidelberg, which is highly experienced and independent of other trial staff [89].

### Data Monitoring and Safety Board (DMSB)

Safety will be strongly monitored, and an independent Data Safety Monitoring Board (DSMB) will be established. All members of the DSMB have experience with RCTs and/or DSMB membership. The DSMB will be informed about adherence to the protocol, patient recruitment, and observed SAEs. The DSMB will receive the corresponding reports at regular intervals (every 6 months).

### Harms

As A-FFIP is a psychotherapeutic intervention, a very low frequency of (S)AEs is expected. Nevertheless, a Data and Safety Monitoring Board (DSMB) will be installed and safety relevant events will be reported to this board. Based on the recommendations of the DSMB, the study might be stopped. All (S)AEs that occur after the subject has signed the informed consent document will be documented on the pages provided in the CRF. SAEs must be reported to the principal investigator within 24 h after the SAE becomes known using the ‘Serious Adverse Event’ form. The investigator must also inform the site monitor in all cases. The investigator is responsible for notification of SAEs to the responsible institutional review board. Patients with SAEs will receive regular medical care in the public health care system.

Patients and their legal representatives will be insured for travel to the way to the regarding study centre (Reference number: F-W20; File number: 2330 991,102; ECCLESIA mildenberger HOSPITAL GmbH).

- Cover in case of death: 50,000 Euro
- Cover in case of invalidity: 100,000 Euro

### Ethics and dissemination

#### Information to the Institutional Review Board (IRB) or Ethical Committees (RECs)

Before the start of the trial, the trial protocol (Additional file 1), informed consent document (Additional file 2), and any other appropriate documents will be submitted to the respective local IRB or the respective Research Ethical Committee (REC). Formal approval by the IRB/REC should preferably mention the title of the trial, the trial code, the trial site, and any other documents reviewed. It must mention the date on which the decision was made and must be officially signed by a committee member. This documentation must also include a list of members of the IRB present on the respective meeting. Before the first subject is enrolled in the trial, all ethical and legal requirements must be met. Neither the investigator nor any person or institution involved in the trial will alter this trial protocol without obtaining the written agreement of the other parties involved. Amendments must be evaluated to determine whether a formal approval must be sought and whether the informed consent document should also be revised. The investigator must keep a record of all communications with the IRB/REC and the regulatory authorities.

### Consent

All participants and their caretakers will be informed about the aims of the trial, its benefits and risks, and written informed consent (Additional file 2) will be obtained from all legal guardians before randomisation or any other study-specific procedure. This will be done by clinicians with longstanding research experience who are familiar with the study protocol and the A-FFIP intervention (psychologists, psychotherapist and physicians). A subgroup of participants from the University Clinic Frankfurt (*N* = 60 at T1) will consent to an additional part (eye-tracking). Participants are allowed to only take part in the intervention study and do not have to agree to the other part (eye-tracking). A copy of the signed informed consent document will be given to the caretaker(s). Participants respectively their caretakers can withdraw from the study any time without giving a reason.

### Dissemination

The study design and results will be presented at national and international conferences (including conferences organised by parent or patient organisations) and published in peer-reviewed journals (open-access planned). Also, the study results will be integrated in the German AWMF S3 clinical guidelines on ASD, Part 2: Therapy. Authorship on dissemination papers will follow ICMJE guidelines and journal requirements. There will be no use of professional writers.

## Supplementary information

**Additional file 1.** Standard Protocol Items: Recommendations for Interventional Trials (SPIRIT) 2013 Checklist: recommended items to address in a clinical trial protocol and related documents.

**Additional file 2.** Model consent form.

## Data Availability

The datasets generated and analysed during the current study are available in the repository of Prof. Dr. Christine M Freitag. It is planned to make the trial data available to additional researchers for re- and meta-analyses after the data (primary/secondary outcomes, predictors/moderators/mediators) have been published by the involved researchers.

## Abbreviations

ADI-R: Autism Diagnostic Interview
ADOS: Autism Diagnostic Observation Schedule
AEs: Adverse events
A-FFIP: Frankfurt Early Intervention Programme for toddlers and preschool children with ASD
ASD: Autism Spectrum Disorder
Bayley-III: Bayley Scales of Infant and Toddler Development – Third Edition
BOSCC: Brief Observation of Social Communication Change
BRIEF-P: Behaviour Rating Inventory of Executive Function-Preschool Version
CBCL1½-5: Child Behaviour Checklist 1½-5
CRF: Case Report Form
C-TRF: Teacher Report Form 1½-5
DASS-21: Depression Anxiety and Stress Scales – short form
DCMA: Dyadic Communication Measure for Autism
DQ: Developmental Quotient
*DSM-5*: *Diagnostic and Statistical Manual of Mental Disorders, version 5*
DSMB: Data and Safety Monitoring Board
DTT: Discrete trial training
EIAU: Early intervention as usual
ESCS: Early Social Communication Scales
ESDM: Early Start Denver Model
fMRI: Functional magnetic resonance imaging
FQOLS-2006 ID/DD: Family quality of Life Survey-2006 ID/DD version
ICH-GCP (E6): International Council for Harmonisation of Technical Requirements for Pharmaceuticals for Human Use – Good Clinical Practice
IQ: Intelligence Quotient
IRB: Institutional Review Board
ISCED: International Standard Classification of Education
KKS: Coordination Centre for Clinical Trials
MMRM: Mixed model for repeated measures approach
NDBI: Naturalistic developmental behavioural interventions
PATCS: Parent Adherence to Treatment and Competence Scale
PSOC: Parenting Sense of Competence Scale
RBS-R: Repetitive Behaviour Scale-Revised
RCT: Randomised controlled trial
SAEs: Serious adverse events
SRS-16: Social Responsiveness Scale – short version
WPPSI-III: Wechsler Preschool and Primary Scale of Intelligence
