# Supplementary material for: Study protocol of the multi-centre, randomised controlled trial of the Frankfurt Early Intervention Programme A-FFIP versus early intervention as usual for toddlers and preschool children with Autism Spectrum Disorder (A-FFIP study)
Source: Trials. 2020 Feb 24;21:217. doi: 10.1186/s13063-019-3881-7 (PMC7038602; doi:10.1186/s13063-019-3881-7)
Supplement: Supplementary file 2 — Additional file 2. Model consent form. [file 13063_2019_3881_MOESM2_ESM.doc]

|  | **STUDY PERIOD** | | | | | | |
| --- | --- | --- | --- | --- | --- | --- | --- |
|  | **Enrolment** | **Baseline** | **Allocation** | **Post-allocation** | | | **Close-out** |
| **TIMEPOINT** | ***T_1_***  ~ -2 weeks | ***T_2_***  ~ -1 week |  | ***T_3_***  + 3 month | ***T_4_***  + 6 month | ***T_5_***  + 9 month | ***T_6_***  + 12 month |
| **ENROLMENT:** |  | | | | | | |
| **Eligibility screen**  (ADI-R, ADOS-2,  cognitive Assessments) | **X** |  |  |  |  |  |  |
| Informed consent | **X** |  |  |  |  |  |  |
| Allocation |  |  | **X** |  |  |  |  |
| **INTERVENTIONS:** |  | | | | | | |
| Frankfurt early intervention program (A-FFIP) |  |  |  |  |  |  |  |
| Early Intervention as usual (EIAU) |  |  |  |  |  |  |  |
| **ASSESSMENTS:** |  | | | | | | |
| ***Primary Objectives*** |  |  |  |  |  |  |  |
| BOSCC |  | **X** |  |  | **X** |  | **X** |
| ***Secondary Objectives*** |  | | | | | | |
| ADOS-2 | **X** |  |  |  | **X** |  | **X** |
| Bayley-III / WPPSI-III | **X** |  |  |  |  |  | **X** |
| SRS-16 (Parents [P]) |  | **X** |  | **X** | **X** | **X** | **X** |
| SRS-16 (Kindergarten [K]) |  | **X** |  |  | **X** |  | **X** |
| RBS-R (P) |  | **X** |  | **X** | **X** | **X** | **X** |
| RBS-R (K) |  | **X** |  |  | **X** |  | **X** |
| CBCL 1 ½-5 (P) |  | **X** |  | **X** | **X** | **X** | **X** |
| C-TRF (K) |  | **X** |  |  | **X** |  | **X** |
| BRIEF-P (P) |  | **X** |  | **X** | **X** | **X** | **X** |
| BRIEF-P (K) |  | **X** |  |  | **X** |  | **X** |
| PSOC (P) |  | **X** |  | **X** | **X** | **X** | **X** |
| DASS-21 (P) |  | **X** |  | **X** | **X** | **X** | **X** |
| FQOLS-2006 IDD (P) |  | **X** |  | **X** | **X** | **X** | **X** |
| ***Mediating Mechanisms*** |  | | | | | | |
| ESCS |  | **X** |  | **X** | **X** | **X** |  |
| DCMA |  | **X** |  | **X** | **X** | **X** |  |
| Bayley-III subscales |  |  |  | **X** | **X** | **X** |  |
| ***Moderating Mechanisms*** |  | | | | | | |
| Eye-Tracking |  | **X** |  |  | **X** |  | **X** |
| ***Adherence and Fidelity*** |  | | | | | | |
| *PATCS* |  |  | **weekly** | | | | |
| *A-FFIP documentation* |  |  | **After every session** | | | | |

Fig 1. Schedule of assessments.

Key: *ADI-R* Autism Diagnostic Interview - Revised *ADOS-2 Autism Diagnostic Observation Schedule BOSCC Brief Observation of Social Communication Change SRS-16 Social Responsiveness Scale – short version RBS-R Repetitive Behavior Scale – Revised CBCL 1 ½-5* Child Behavior Checklist 1 ½-5 *C-TRF 1 ½-5 BRIEF-P Behavior Ratin Inventory of Executive Function-Preschool version PSOC Parent sense of competence scale DASS-21 Depression Anxiety and Stress Scale – short form FQOLS Family quality of Life Survey ESCS Early Social Communication Scale DCMA Diadic Communication Measure for Autism Bayley-III Bayley Scales of Infant and Toddler Development 3^rd^ Edition PATCS Parent Adherence to treatment and Competence Scale*
